# Distribution of *COL8A2* and *COL8A1* gene variants in Caucasian primary open angle glaucoma patients with thin central corneal thickness

**Published:** 2010-10-29

**Authors:** T. Desronvil, D. Logan-Wyatt, W. Abdrabou, M. Triana, R. Jones, S. Taheri, E. Del Bono, L.R. Pasquale, M. Olivier, J.L. Haines, B.J. Fan, J.L. Wiggs

**Affiliations:** 1 Department of Ophthalmology, Harvard Medical School and Massachusetts Eye and Ear Infirmary, Boston, MA; 2 Rosalind Franklin University of Medicine and Science, Chicago, IL; 3 Center for Human Genetics Research, Vanderbilt Medical School, Nashville TN

## Abstract

**Purpose:**

One approach to identify genes that contribute to common complex ocular disorders such as primary open angle glaucoma (POAG) is to study the genetic determinates of endophenotypes that are defined by underlying pre-disposing heritable quantitative traits such as central corneal thickness (CCT). Collagen VIII is a major component of Descemet’s membrane and studies in mice have indicated that targeted inactivation of the genes encoding the collagen type 8 alpha1 (Col8a1) and collagen type 8 alpha2 (Col8a2) subunits (*COL8A1* and *COL8A2*) results in thinning of the corneal stroma and of Descemet’s membrane. The purpose of this study is to evaluate *COL8A1* and *COL8A2* as candidate genes for thin CCT in human POAG patients.

**Methods:**

100 Caucasian POAG patients were enrolled in this study. The entire *COL8A1* and *COL8A2* coding sequence was determined in 8 patients with CCT<513 µm (one standard deviation (36 microns) below the mean (550 microns) and 8 patients with CCT>586 µm (one standard deviation above the mean). Selected *COL8A2* exons containing variants of interest were sequenced in the full POAG cohort. Association and quantitative trait analyses were performed.

**Results:**

Three patients with CCT less than 513 µm and advanced POAG were found to have missense changes in *COL8A2*; two patients had a previously identified mutation, R155Q and one had a novel change, P678L (p=0.0035, Fisher’s exact test). Missense changes were not found in any of the patients with CCT>513 µm and missense changes in the *COL8A1* gene were not found in any patient. One common *COL8A2* SNP, rs274754 was also statistically associated with CCT (p=0.018).

**Conclusions:**

In this study we have identified *COL8A2* missense changes in a group of Caucasian patients with very thin CCT and advanced POAG. These results suggest that DNA sequence variants in the *COL8A2* gene may be associated with thin corneas in some glaucoma patients. Further study of *COL8A2* variants in other patient populations, especially those with thinner CCT such as African-Americans would provide further support for a role of *COL8A2* in corneal thickness and in glaucoma.

## Introduction

Primary open angle glaucoma (POAG) is phenotypically and genetically complex. One approach to identify genes that contribute to common complex traits is to study the genetic determinants of endophenotypes that are defined by underlying pre-disposing quantitative traits [1]. Mapping genes influencing the related quantitative trait, rather than the complete complex phenotype, has several important advantages including objective phenotype definitions and a possible reduction in the underlying molecular heterogeneity. Several quantitative traits with significant heritability are associated with POAG including intraocular pressure (IOP), optic nerve vertical cup-to-disc ratio, optic nerve area, and central corneal thickness (CCT) [2-9].

The ocular hypertension treatment trial (OHTS) initially identified thin CCT as a risk factor for progression from ocular hypertension to glaucoma [10]. Subsequently, other studies have suggested that thin CCT is associated with increased severity of visual field loss and more rapid progression of visual field loss [11-15]. Central corneal thickness is a normally distributed, highly heritable quantitative trait in human populations [16], with individuals of African race having lower CCT than Caucasian populations [17,18]. The increased incidence of POAG in African populations is consistent with the increased risk of disease associated with thin CCT.

Candidate gene studies and a recent genome-wide association study have identified several genes that may contribute to CCT variation, including the genes for type I collagen [19] and the genes coding for collagen 5 alpha1 (*COL5A1*), autogenous vein graft remodeling associated protein 8 (*AVGR8*), and A-kinase anchor protein 13 (*AKAP13*) [20]. These results suggest that genes coding for proteins that maintain corneal stromal integrity may be good candidates for genetic determinants influencing the trait.

Collagen VIII (COL8) is a major component of Descemet’s membrane, and is composed of two subunits, collagen VIIIA1 (COL8A1) and collagen VIIIA2 (COL8A2) which form homotrimers [21]. Targeted inactivation of the *COL8A1* and *COL8A2* genes in mice results in anterior segment dysgenesis and thin corneal stroma [22], suggesting that *COL8A1* and/or *COL8A2* may contribute to the development of thin CCT. Further support for a role of *COL8A2* in corneal thickness comes from a more recent study documenting thin corneas in mice with a *COL8A2* missense change, G257D [23]. The purpose of this study is to evaluate *COL8A1* and *COL8A2* as candidate genes for thin CCT in human POAG patients.

## Methods

### Patients

This study was approved by the institutional review board of the Massachusetts Eye and Ear Infirmary, Boston, MA. After informed consent, 100 Caucasian POAG patients from the Massachusetts Eye and Ear Infirmary glaucoma service were enrolled in this study. Central corneal thickness (CCT) was measured using an ultrasonic pachymeter (DGH Technology, Inc., Exton, PA). The recorded value was an average of three measurements for each eye. The number used for CCT for each patient was the average of each eye. The following criteria were used to establish POAG affected status: 1) IOP≥22 mmHg in both eyes on 2 occasions, or IOP≥19 mmHg in both eyes on treatment with 2 or more glaucoma medications; 2) Visual field loss in at least one eye on a reliable visual field (reliable visual field is defined by fixation loss ≤33%, false positive rate ≤20% and false negative rate ≤20%) that is in a distribution consistent with nerve fiber layer loss and corresponds to changes in the optic nerve; and 3) Optic nerve damage in at least one eye characterized by *two* of the following: vertical cup/disc ratio >0.7, superior or inferior neuroretinal rim <0.1, focal notching of the superior or inferior neuroretinal rim, nerve fiber bundle defect with a width of 2 or more retinal vein diameters located 1 disc diameter from the optic nerve, asymmetry of the cup/disc ratio >0.2 without asymmetric refraction, and disc hemorrhage. Patients with known corneal disease and patients who had undergone corneal surgery, including refractive surgery, were excluded from this study.

### DNA sequencing

Genomic DNA was prepared from buccal cell samples using established techniques (Gentra, Minneapolis, MN). Initially, the entire coding region of *COL8A1* and *COL8A2* was sequenced in 8 patients with CCT<513 μm (one standard deviation from the mean of 550±36 μm) and 8 patients with CCT>586 μm (one standard deviation from the mean). Genomic DNA was sequenced using primers (Table 1) designed to amplify the coding exons as well as the adjacent splice sites for both the *COL8A1* and *COL8A2* genes.  PCR was performed in a thermal cycler (model 2720; Applied Biosystems Inc., Foster City, CA) set at the following parameters: 50 °C for 2 min, 95 °C for 10 min, 92 °C for 15 s, and 58 °C for 1 min for a total of 60 cycles. PCR products were directly sequenced on the ABI PRISM 3100 Genetic Analyzer (Applied Biosystems) with BigDye Terminators (Applied Biosystems) according to standard protocols. Selected *COL8A2* exons containing variants of interest were sequenced in the entire POAG cohort using the same techniques.

**Table 1:** Characteristics of primers used for amplification and sequencing.

**Primer name**	**Sequence**	**Melting temp**	**Product size**
**Primers for coding exons of *COL8A1* (NM_001850)**			
Exon 4 Forward	AAGTCACTTGGCCTTGCAG	59.02	456
Exon 4 Reverse	CCCCTCTGATCCCATAATTTAG	58.84	
Exon 5_1 Forward	ACTTCATTGATGTGAGAGACAATC	57.34	630
Exon 5_1 Reverse	TGGAGCCCCTGGCTTTC	62.33	
Exon 5_2 Forward	AGGTGCGCCAGGTGTAAAG	61.24	594
Exon 5_2 Reverse	ACTTCACCAAGGAAACCTGG	59.04	
Exon 5_3 Forward	CAAAGGAGAAGGTGGGATTG	59.52	584
Exon 5_3 Reverse	TGCCTTTCTTAGCCCCGTAG	61.59	
Exon 5_4 Forward	GAGTGGCAGGACTTCATGG	59.20	777
Exon 5_4 Reverse	TGTACACAATGGTCCAAATTTTC	58.78	
**Primers for coding exons of *COL8A2* (NM_005202)**			
Exon 1 Forward	CAGGGCTGGCTTGATGAC	60.36	312
Exon 1 Reverse	AGGGAGGCAGGGGATTTG	62.33	
Exon 2_1 Forward	GGAATGGGTAGATGGGGTC	59.01	599
Exon 2_1 Reverse	AACCAGGTTTGCCTAAGCC	59.18	
Exon 2_2 Forward	GATAATGGAGTGGGCCAGC	60.44	580
Exon 2_2 Reverse	CACTAGGCCCCTGGTCAC	59.05	
Exon 2_3 Forward	GCTTCCTGGCAGACGTG	59.00	597
Exon 2_3 Reverse	AGCCCAAACTGTGGCTTG	59.83	
Exon 2_4 Forward	CTCCCCTGGAATCACGG	59.98	636
Exon 2_4 Reverse	TTGAAAAGGTCGCTCTACCAC	59.37	

### Statistical methods

The association of missense changes in *COL8A2* and CCT<513 µm was assessed using Fisher’s exact test. Quantitative trait analysis for rs274754 and rs3738360 was performed using PLINK (version 1.07) [24], and p-values were adjusted for gender and age of enrollment using logistic regression.

## Results

### Identification of sequence variants in *COL8A1* and *COL8A2*

**Figure 1:**
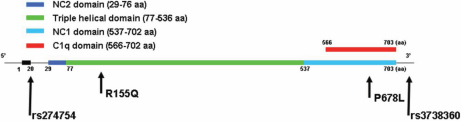
Structure of the COL8A2 gene and protein domains. The *COL8A2* gene consists of two exons separated by single intron. The protein contains NC2, NC1, C1q, and triple helical domains as indicated. The locations of the variants studied are indicated with arrows.

100 Caucasian POAG patients with a mean CCT of 550±36 µm (range 488–676 µm) were evaluated for this study. The mean CCT in our POAG sample is similar to that observed in the normal Caucasian population [25]. The mean age at enrollment was 68 years and the population was 51% female. Initially we sequenced the entire coding sequence for both the *COL8A1* and *COL8A2* genes in 8 patients with CCT<513 µm (one standard deviation from the mean) and in 8 patients with CCT>586 µm (one standard deviation from the mean). Three patients with CCT less than 513 µm were found to have missense changes in *COL8A2*; two patients had a previously identified mutation, R155Q and one had a novel change, P678L. Missense changes were not found in any of the patients with CCT>586 µm and missense changes were not found in any patients in the *COL8A1* gene. Two common *COL8A2* SNPs, rs274754 and rs3738360 were also found in both groups of patients. The entire cohort was further genotyped for R155Q, P678L, rs274754, and rs3738360 by genomic sequencing. Missense changes were not identified in any of the remaining POAG patients. The location of each variant in the *COL8A2* gene is shown in Figure 1.

### Quantitative trait analysis

The two common *COL8A2* SNPs (rs274754 and rs3738360) were genotyped in the entire cohort and analyzed for association with CCT. The genotype frequencies, mean CCT values for each genotype and associated p values for these two SNPs are shown in Table 2. SNP rs274754, located in the first intron is statistically associated with CCT (p=0.018), while SNP rs3738360, located in the 3′ UTR does not demonstrate an association in this group of individuals.

**Table 2:** QTL association analysis of common SNPs and CCT in Caucasian POAG patients.

**SNP**	**Genotype**	**N**	**CCT µm (mean±SD)**
rs3738360	TT	78	550.6±38.0
	GT	14	545.5±30.9
	GG	0	-
	Total	92	p-trend=0.64; p-adj=0.57
rs274754	TT	45	561.3±32.4
	CT	23	539.9±30.4
	CC	18	545.1±48.8
	Total	86	p-trend=0.047; p-adj=0.018

CCT: the average measures of central corneal thickness between left and right eyes; p-adj: p value adjusting for sex and age of enrollment.

### *COL8A2* missense carrier phenotypes

Phenotypic information for the three *COL8A2* missense carriers is shown in Table 3. All three patients have very thin CCT, and advanced POAG including elevated intraocular pressure, optic nerve degeneration, and significant visual field defects. None of these patients had undergone refractive surgery or any other type of corneal surgery before CCT measurement.

**Table 3:** *COL8A2* missense carrier phenotypes.

** **	**Patient ID (genotype)**		
**Phenotype information**	**4381 (R155Q)**	**4964 (R155Q)**	**4995 (P678L)**
Gender	Male	Male	Male
CCT µm (OD, OS)	502, 526	484, 481	507, 497
Age of enrollment	49	86	73
Visual acuity (OD, OS)	20/25, 20/50	20/60, 20/30	20/25, 20/20
Refractive error (OD, OS)	-1.00 -0.75 x002 -1.50 -1.25 x017	-1.75 -1.26 x065 0.00 -2.25 x098	-0.50 -0.50 x150 0.00 -0.50 x047
Gonioscopy	Open angle	Open angle	Open Angle
IOP >21	Yes	Unknown	Yes
Optic nerve VCDR (OD, OS)	0.95, 0.95	0.95, 0.90	0.9, 0.8
Visual Fields*	OD: PSD 11.5- NS (S/I), ND (S/I), PS (S) OS: PSD 9.1- NS (S/I), ND (S/I), PS (S/I)	OD: Central island only OS: Central island only	OD: PSD 9.5- NS (S/I), ND (S/I), PS (S/I) OS: PSD 5.5- ND (S), PS (S/I)

*Humphrey automated visual fields (24–2) were obtained for individuals 4831, 4995, and Goldman visual fields were available for individual 4964. Abbreviations: VCDR- vertical cup to disc ratio; PSD- pattern standard deviation; NS- Nasal step; ND- Nasal depression; PS- paracentral scotoma; S- superior; I- inferior.

### Functional significance of *COL8A2* missense variants

**Figure 2:**
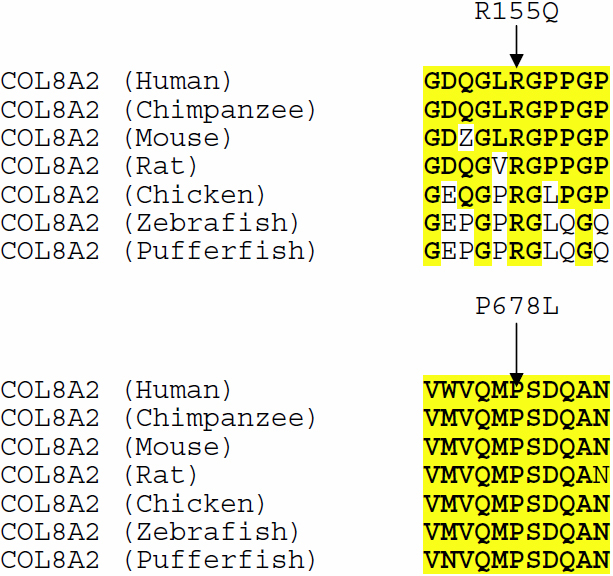
Evolutionary conservation of R155Q and P678L. Arrows indicate the position of Arginine 155 and Proline 678. Conserved amino acids are highlighted in yellow.

R155Q and P678L were found in 3 of 16 patients with CCT<513 µm and in none of the patients with CCT>513 µm (p=0.0035, Fisher’s exact test). Both R155Q and P678L are evolutionarily conserved amino acids (Figure 2). R155Q has been previously defined as a pathological variant contributing to Fuch’s Endothelial Dystrophy (FED) [26]. To further assess the probability that these variants impact corneal pathogenesis we used three tests of functional significance: PolyPhen-2 [27], SIFT [28] and PMUT [29]. Both PolyPhen-2 and PMUT identified P678L as a pathological variant (Table 4), while SIFT indicated that the proline to leucine change at position 678 could be tolerated. Surprisingly, both PMUT and SIFT suggested that R155Q, previously implicated in FED and PPMD, could be a tolerated variant while PolyPhen-2 was not able to make an assessment.

**Table 4:** Tests of functional significance.

**SNP**	**PolyPhen-2**	**PMUT**	**SIFT**
R155Q	Unable to assess	Neutral (score=0.439)	Tolerated
P678L	Probably damaging (score=0.999)	Pathological (score=0.845)	Tolerated

## Discussion

In this study we have identified *COL8A2* missense changes in a group of Caucasian patients with very thin CCT and advanced POAG. R155Q was found in two patients and P678L was found in one patient with CCT<513 µm. Both of these missense changes are evolutionarily conserved and are likely to be pathogenic. R155Q is located in the evolutionarily conserved triple helical domain. Although tests of functional significance suggest that the glutamine could be tolerated at position 155, the R155Q change has been previously associated with Fuch’s endothelial dystrophy (FED) [26] suggesting that the variant has a role in corneal disease. P678L is a novel *COL8A2* missense change that is located in the highly conserved C1q domain. This variant is predicted to be pathologic by PolyPhen-2 and PMUT. Other *COL8A2* missense changes have also been found in FED (R34H, R304Q, Q455K, Q455V, and L450W) [26,30-33]. *COL8A2* mutations have not been found in patients with keratoconus or keratoglobus [34]. Our patients did not have clinical evidence of any of these corneal disorders.

As the previous reports relating *COL8A2* missense changes to corneal disease have not included CCT measurement as part of the clinical assessment, it is not possible to determine if the patients carrying these gene variants also had thin CCT. However, given our results and the complex genetics of FED [35], it is likely that *COL8A2* missense changes are responsible for thin CCT and that *COL8A2* variants may also be one factor that can contribute to FED. Indeed several reports support this hypothesis, including a report describing familial aggregation, but not perfect segregation, of a *COL8A2* missense change and FED [36] and the identification of the R155Q change in one FED affected patient and two controls in a population from South India [37]. The R155Q variant is also relatively common in the Japanese, a population known to have thinner CCT [38,39].

We also evaluated two common SNPs, rs274754 located within intron 1 near the 5′ end of the gene and rs3738360 located in the 3′UTR. Only SNP rs274754 was statistically associated with CCT, with the G allele associated with thinner CCT. Interestingly the G allele of rs274754 is more common in Africans (dbSNP), and individuals of African origin have thinner corneas and a higher incidence of POAG than Caucasians [40,41]. It would be of interest to evaluate the association between CCT and rs274754 and other common *COL8A2* SNPs in a larger POAG sample and also in African populations.

POAG is inherited as a complex non-Mendelian trait that is likely to result from multiple genetic and environmental factors. Identifying genes responsible for POAG pre-disposing endophenotypes, such as thin CCT, is one path toward defining the underlying complex genetic architecture of the disease. This study suggests that *COL8A2* gene variants can contribute to thin CCT. Further studies identifying genes that contribute to POAG endophenotypes will help define the molecular events underlying the complex phenotype as well as lead to gene-based tests for screening and diagnosis.
